# Promotion of gastric tumor initiating cells in a 3D collagen gel culture model via YBX1/SPP1/NF-κB signaling

**DOI:** 10.1186/s12935-021-02307-x

**Published:** 2021-11-10

**Authors:** Shuangya Deng, Lun Li, Shu Xu, Xiaobo Wang, Tong Han

**Affiliations:** Department of General Surgery, The Second Xiangya Hospital of Central South University, Changsha, China

**Keywords:** Tumor initiating cells (TICs), Collagen, Integrin β1 signaling, Y-box binding protein 1 (YBX1), Secreted phosphoprotein 1 (SPP1), Gastric cancer

## Abstract

**Background:**

The high potential for tumor recurrence and chemoresistance is a major challenge of clinical gastric cancer treatment. Increasing evidence suggests that the presence of tumor initiating cells (TICs) is the principal cause of tumor recurrence and chemoresistance. However, the underlying mechanism of TIC development remains controversial.

**Methods:**

To identify novel molecular pathways in gastric cancer, we screened the genomic expression profile of 155 gastric cancer patients from the TCGA database. We then described an improved 3D collagen I gels and tested the effects of collagen on the TIC phenotype of gastric cells using colony formation assay, transwell assay, and nude mouse models. Additionally, cell apoptosis assay was performed to examine the cytotoxicity of 5-fluorine and paclitaxel on gastric cancer cells cultured in 3D collagen I gels.

**Results:**

Elevated expression of type I collagen was observed in tumor tissues from high stage patients (stage T3–T4) when compared to the low stage group (n=10, stage T1–T2). Furthermore, tumor cells seeded in a low concentration of collagen gels acquired TIC-like phenotypes and revealed enhanced resistance to chemotherapeutic agents, which was dependent on an integrin β1 (ITGB1)/Y-box Binding Protein 1 (YBX1)/Secreted Phosphoprotein 1 (SPP1)/NF-κB signaling pathway. Importantly, inhibition of ITGB1/NF-κB signaling efficiently reversed the chemoresistance induced by collagen and promoted anticancer effects in vivo.

**Conclusions:**

Our findings demonstrated that type I collagen promoted TIC-like phenotypes and chemoresistance through ITGB1/YBX1/SPP1/NF-κB pathway, which may provide novel insights into gastric cancer therapy.

**Supplementary Information:**

The online version contains supplementary material available at 10.1186/s12935-021-02307-x.

## Background

Gastric cancer is the fifth most common carcinoma and the third leading cause of cancer associated death worldwide [1]. It is known to be a highly heterogeneous disease, which is mainly treated with surgical resection combined with perioperative or adjuvant chemotherapy. Despite the advance of clinical interventions, many patients suffer from recurrent disease and the development of drug resistance after resection and adjuvant treatment. In metastatic or recurrent gastric cancer, patient outcomes are poor, with median survival being about 12 months. Gastric cancer has a high potential for recurrence, which is mostly due to residual tumor initiating cells (TICs) after treatment [2]. However, the underlying mechanism of TIC-induced tumor progression is still poorly understood, and there is an urgent demand to develop innovative therapeutic strategies to eliminate TICs and improve outcomes in gastric cancer treatment.

TICs, also called cancer stem cells or tumor repopulating cells, are a small fraction of cells within tumor tissues, which largely contribute to both tumor recurrence as well as drug resistance in cancer-associated disease [3]. Recently, gastric TICs have been demonstrated to be tightly correlated with clinical tumor relapse and drug resistance during gastric cancer therapy [4]. A series of specific cellular surface markers, including CD133, CD24 and CD44, have been utilized to distinguish TICs from bulk gastric tumor cells [5–7]. As previously reported, the expression of CD44 was demonstrated to efficiently indicate tumor burden and metastatic potential in gastric patients [8]. Elevated expression of CD44 protein is also linked to poor prognosis and distant metastasis in human gastric cancer, indicating the essential roles of TICs in gastric cancer progression [9]. The presence of TICs has been reported in diverse studies [10, 11]; however, the concept of TICs still remains controversial. Increasing evidence suggests that differentiated cancer cells, such as CD133 negative melanoma cells, are capable of forming subcutaneous tumors in immunodeficient mice [12]. More importantly, specific environmental cues, such as 3D fibrin culture or serum-free suspension culture, could equip differentiated tumor cells with stem-like characteristics and facilitate tumorigenicity of cancer cells in nude mice [13]. These findings infer that tumor cells might be able to obtain the TIC phenotype under specific conditions and there is a dynamic conversation between TICs and bulk tumor cells [14].

Increasing studies provide evidence to highlight the influence of the tumor microenvironment on TIC regulation [15, 16], which gives rise to tumorigenicity and drives tumor heterogeneity. The extracellular matrix, along with the stromal cells and sequestered cytokines, serves as the major component of microenvironment, and together offer extracellular cues and regulate the TIC phenotype. The extracellular matrix around tumor cells is a three-dimensional structure, which consists of collagens, fibronectin and glycoproteins to organizes cells within tissues. Studies have suggested that compounds in the extracellular matrix, such as collagen, laminin and fibronectin, are capable of supporting cancer cell proliferation and regulating TIC/cancer stem cell maintenance [17, 18]. Consistently, 3D Matrigel or fibrin gels have been shown to mediate the TIC phenotype in diverse tumor cells through extracellular matrix binding to integrin receptors [19], which further triggers the activation of pro-survival signaling pathways. However, the specific correlation between extracellular matrix-associated compounds and gastric TICs remains poorly understood, and there is still lack of evidence demonstrating how TICs participate in gastric cancer progression, contributing to poor outcomes in patients.

To identify novel molecular pathways in gastric cancer, we screened profile changes in gene expression and found a dramatic upregulation of collagen-associated genes in gastric tumor tissues from patients. We describe an improved 3D collagen culture model and provide evidence to suggest that gastric cancer cells are plastic and capable of acquiring the TIC phenotype in the presence of collagen. Mechanistically, we demonstrated that type I collagen promotes TIC proliferation through ITGB1/YBX1/SPP1/NF-κB signaling. Furthermore, we used a combination of ITGB1 and NF-κB pathway inhibitors to eliminate TICs and reverse drug resistance, representing an innovative therapeutic avenue for gastric cancer therapy.

## Materials and methods

### Cell culture and reagents

Human gastric cancer cell lines SGC-7901 and BGC-823 were purchased from American Type Culture Collection (ATCC) and maintained in RPMI 1640 complete culture medium (Gibco, USA) supplemented with 10% fetal bovine serum (Hyclone, USA). Chemotherapeutic 5 fluorine (5-FU) and paclitaxel (PTX) were purchased from Sigma (USA). NF-κB inhibitor JSH-23 and ITGB1 inhibitor LDV were purchased from MedChemExpress (USA). The ITGB1, SPP1 and YBX1 knockdown SGC-7901/BGC-823 cells were purchased from Cyagen (China) and the knockdown efficiency was determined by western blotting. All other reagents were purchased from Biyuntian (China) or Thermo fisher (USA) and of analytical grade.

### Clinical specimens

Paraffin sections of human gastric tumor tissues were obtained from the pathology department of Hospital of Central South University. All tumor tissues were divided into high stage group (n = 10, stage T3–T4) and low stage group (n = 10, stage T1–T2), according to the Paris Typing of Gastric Cancer. 20 patients were informed with written consent and agreed to participated in the study. The clinical experiments were performed according to the guidance of the Declaration of Helsinki and approved by the Ethics Committee of the Hospital of Central South University.

### Establishment of 3D collagen culture system

For 3D collagen culture of gastric cancer cells, type I collagen solution (Solarbio, China) was diluted to 0.5, 1, and 2 mg/ml with culture medium. 2 × 10^4^ tumor cells were added into 300 µl collagen solution. Then 30 µl NaOH (1 M) was added into the tumor cells/collagen solution and mixed thoroughly. The 330 µl collagen solution was seeded into 48 well plate and cultured in a 37 °C incubator. After 6 h of incubation, the solid clotty collagen gels were removed into RPMI 1640 complete medium supplemented with 10% fetal calf serum for further culture. The tumor cells in 3D clotty collagen gels were pictured every day and the colony sizes were determined by image J 2.0 software. After 5 days, type I collagenase (Sigma, USA) was added into the culture medium to collect gastric cancer cells for further analysis.

### Colony formation assay

Five hundred single SGC-7901 or BGC-823 cells were immediately seeded in 0.66% solidified agar-based 6-well plate and cultured with 1640 RPMI complete culture medium for 14 days. The emerging colonies were fixed with 4% paraformaldehyde and stained with crystal violet. The colonies were pictured and counted for further analysis.

### Transwell assay

10^5^ SGC-7901 and BGC-823 cells were seeded in transwell insert (8 μm, Corning, USA) containing 100 µl culture medium (10% FBS). The bottom chamber was filled with 500 µl culture medium containing 20% FBS. After 24 h culture, the migrating cells were fixed with paraformaldehyde and stained with crystal violet. Relative migrating cells were count and each experiment was performed three times independently.

### Cell apoptosis assay

Cell apoptosis was determined using the FITC-Annexin V/PE-PI apoptosis detection kit (Becton Dickinson, USA) according to the guidance of manufacturer. Briefly, SGC-7901 and BGC-823 cells were treated with 5-FU (1 µg/ml) or PTX (2 µg/ml) for 48 h. Then cells were stained with FITC-Annexin V/PE-PI staining solution for 15 min. Cell apoptosis was detected by a C6 flow cytometer (Becton Dickinson, USA).

### Real-time quantitative PCR

Total RNA of SGC-7901 or BGC-823 cells was extracted using Total RNA Extraction Kit (Solarbio, China). The quantification of mRNA was performed by real-time PCR using SYBR green dye (Thermo, MA, USA). GAPDH was used for normalization. The primers used are listed as follows: human GAPDH forward primer 5′-GGAGCGAGATCCCTCCAAAAT-3′, reverse primer 5′-GGCTGTTGTCATACTTCTCATGG-3′; human integrin β1 forward primer 5′-CCTACTTCTGCACGATGTGATG-3′, reverse primer 5′- CCTTTGCTACGGTTGGTTACATT-3′; integrin β2 forward primer 5′- TGCGTCCTCTCTCAGGAGTG-3′, and reverse primer 5′-GGTCCATGATGTCGTCAGCC-3′; integrin β3 forward primer 5′-GTGACCTGAAGGAGAATCTGC-3′, and reverse primer 5′-CCGGAGTGCAATCCTCTGG-3′; integrin β4 forward primer 5′-GCAGCTTCCAAATCACAGAGG-3′, and reverse primer 5′-CCAGATCATCGGACATGGAGTT-3′; integrin β5 forward primer 5′-TCTCGGTGTGATCTGAGGG-3′, and reverse primer 5′-TGGCGAACCTGTAGCTGGA-3′; integrin β6 forward primer 5′-TCCATCTGGAGTTGGCGAAAG-3′, and reverse primer 5′-TCTGTCTGCCTACACTGAGAG-3′; integrin β7 forward primer 5′-AGAATGGCGGAATCCTCACCT-3′, and reverse primer 5′-TGAAGTTCAGTTGCTTGCACC-3′; integrin β8 forward primer 5′-ACCAGGAGAAGTGTCTATCCAG-3′, and reverse primer 5′-CCAAGACGAAAGTCACGGGA-3′. SOX2 forward primer 5’-GCCGAGTGGAAACTTTTGTCG-3′ and reverse primer 5′-GGCAGCGTGTACTTATCCTTCT-3′; c-Myc forward primer 5′-GGCTCCTGGCAAAAGGTCA-3′ and reverse primer 5′-CTGCGTAGTTGTGCTGATGT-3′; KlF4 forward primer 5′-CCCACATGAAGCGACTTCCC-3′ and reverse primer 5′-CAGGTCCAGGAGATCGTTGAA-3′; Nanog forward primer 5′-TTTGTGGGCCTGAAGAAAACT-3′ and reverse primer 5′-AGGGCTGTCCTGAATAAGCAG-3′; Oct4 forward primer 5′-CTGGGTTGATCCTCGGACCT-3′ and reverse primer 5′-CCATCGGAGTTGCTCTCCA-3′.

### Western blotting

Gastric tumor cells were lysed by 1% NP40 buffer (50 mM Tris-HCl, pH 7.4, 150 mM NaCl, 0.1% SDS) containing protease inhibitor cocktail (Biyuntian, China). Protein quantification was performed using Protein Quantitative Analysis kit (Biyuntian, China). 20 µg protein was separated in 10% SDS gels and transferred to polyvinylidene fluoride Immobilon-membranes. Then protein samples were blocked with 5% Nonfat-Dried Milk and incubated with primary antibodies: anti-ITGB1 (1:1000, Abcam, UK), anti-SPP1 (1:1000, Abcam, UK), anti-YBX1 (1:1000, Abcam, UK), anti- NF-κB (1:1000, Abcam, UK) and anti-β-actin (1:1000, Abcam, UK). Samples were then incubated with HRP-conjugated secondary antibody (1:1000, Thermo fisher, USA) for 1 h at room temperature. The target proteins were visualized using the ECL detection kit (Thermo fisher, USA).

### Immunohistochemistry/immunofluorescence staining

Paraffin sections were dewaxed, and treated with sodium citrate antigen retrieval. Then tissue sections were blocked with 5% goat serum in PBST for 30 min at room temperature, and incubated with primary antibodies: anti-type I collagen antibody (1:200, Abcam, UK), anti-ITGB1 antibody (1:300, Abcam, UK) and anti-NF-κB (1:500, Abcam, UK) for overnight at 4 °C. For immunohistochemistry, samples were then incubated with goat anti-rabbit secondary antibodies (Abcam, UK) and stained with DAPI (Solarbio, China). For immunofluorescence, samples were then incubated with HRP labeled goat anti-rabbit secondary antibodies (Abcam, UK) and stained with hematoxylin (Solarbio, China). The intensity of protein expression was analyzed by image pro plus 6.0 software (USA).

### Flow cytometry

The anti-CD44 antibody (eBioscience, USA) was used for flow cytometry analysis according to the guidance of manufacturer. Samples were analyzed using C6 flow cytometry (Becton Dickinson, USA) and data was analyzed by FlowJo software (TreeStar, USA).

### Animal protocols

Following the approval of the Animal Ethics Committee of Hospital of Central South University, NOD-SCID mice were purchased from Huafukang (China). For tumorigenic assay, 10^5^ SGC-7901 or BGC-823 cells were subcutaneously injected into NOD-SCID mice (n = 10 in each group). Mice were monitored every day until 30 days at the end point. The tumor diameter > 1 mm were determined as tumorigenicity. The percentage of mice with tumorigenicity in 10 mice was determined as tumorigenesis rate.

For tumor volume and survival analysis, mice were subcutaneously injected with 10^6^ dish/3D cultured SGC-7901 cells (n= 6 in each group). After two weeks, mice were treated with PBS, LDV (2.5 mg/kg), PTX (5 mg/kg), JSH-23 (2.5 mg/kg), 5-FU (10 mg/kg) or combining therapy every three days. Tumor volume and survival of mice was recorded every day. The calculation formula of tumor volume is: tumor volume = length × width^2^/2. The animal studies were conducted in accordance with the Public Health Service Policy and complied with the WHO guidelines for the humane use and care of animals.

### Statistical analysis

Gastric cancer patients’ information was downloaded from http://ualcan.path.uab.edu/index.html and https://www.cbioportal.org/. All experiments in our study were conducted three independent times. Data was presented as the mean ± SEM and analyzed by GraphPad 7.0 or SPSS 3.0 software. Statistical significance between groups was calculated by Student’s t-test for two groups, or by one-way ANOVA for three or more groups. The survival rates were evaluated by Kaplan–Meier survival analysis. * means p < 0.05; ** means p < 0.01; ns means no significant difference.

## Results

### 3D collagen gel culture promotes the TIC phenotype in gastric cancer cells

**Fig. 1:**
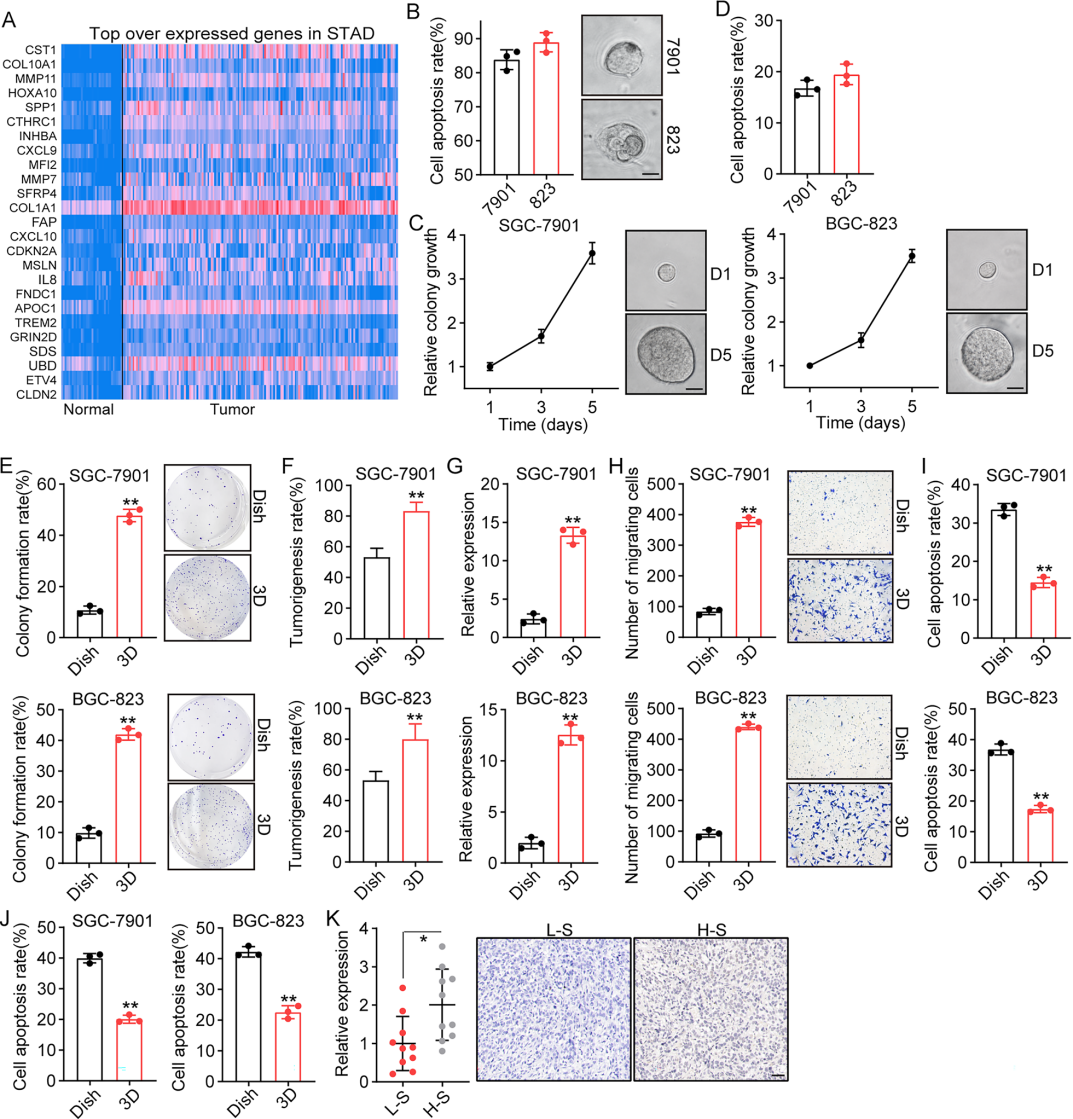
3D collagen gels culture promoted TICs phenotypes of gastric cancer cells. **A** Top overexpression genes in stomach adenocarcinoma (STAD) tissues (n = 156) compared to normal tissues (n = 34) from TCGA database. **B** The presentative image and cell apoptosis of SGC-7901/BGC-823 in collagen gels (1–2 mg/ml) on day5. The scale bar is 50 μm. **C** The colony growth curve of SGC-7901/BGC-823 in collagen gels (0.5 mg/ml). The scale bar is 50 μm. D, the cell apoptosis of SGC-7901/BGC-823 in collagen gels (0.5 mg/ml) on day 5. **E** The colony formation capability of SGC-7901/BGC-823 cultured in dish or 3D collagen gels. **F** The tumorigenic capability of SGC-7901/BGC-823 cultured in dish or 3D collagen gels. 10^5^ SGC-7901 or BGC-823 cells were subcutaneously injected into NOD-SCID mice (n = 10 in each group, day 30). The tumor diameter > 1 mm were determined as tumorigenicity. The percentage of mice with tumorigenicity in 10 mice was determined as tumorigenesis rate. **G** The CD44 expression of SGC-7901/BGC-823 cultured in dish or 3D collagen gels, which was determined by flow cytometry. **H** Cell migration of SGC-7901/BGC-823 cultured in dish or 3D collagen gels (24 h), which was determined by transwell assay. **I** SGC-7901/BGC-823 were cultured in dish or 3D collagen gels for 5 days, then treated with 5-FU (1 µg/ml) for 48 h. The cell apoptosis was examined using Annexin/PI staining. **J** SGC-7901/BGC-823 were cultured in dish or 3D collagen gels for 5 days, then treated with PTX (2 µg/ml) for 48 h. The cell apoptosis was examined using Annexin/PI staining. **K** Immunohistochemical staining of collagen I in gastric tumor tissues from high stage (H-S, stage T3–4) and low stage (L-S, stage T1–2) patients (n = 10 in each group). The scale bar is 50 μm

As reported previously, tumor cells with unique genomic expression profiles frequently display proliferative and TICs characteristics, revealing the ability to drive tumor progression and chemoresistance [20]. To elucidate the underlying mechanism of gastric cancer progression, we screened the genomic expression profile of gastric tumor tissues from the TCGA database and determined the most highly overexpressed genes in tumor tissues (Fig. 1A). Notably, elevated expression collagen-associated genes, including COL10A1 and COL1A1, was observed in tumor tissues when compared to normal tissues. In fact, collagen serves as the major component of the extracellular matrix, and is involved in tumor progression and promoting the TIC phenotype in breast cancer. Herein, to explore the role of collagen in gastric cancer development, gastric cancer cell lines SGC-7901 and BGC-823 cells were seeded in diverse concentrations of 3D collagen gels. Importantly, traditional 3D collagen gels (1 mg/ml–2 mg/ml collagen concentration) used in our study elicited apoptosis in tumor cells (Fig. 1B), whereas a low concentration of collagen (0.5 mg/ml, solid clotty morphology) was able to promote the colony formation of SGC-7901/BGC-823 cells, without obvious cell death (Fig. 1C, D). Therefore, the improved soft 3D collagen gels (0.5 mg/ml) were considered as the ideal concentration for gastric cancer culture.

Compelling studies have implicated that 3D gel culture, such as fibrin gels and Matrigel, can promote the TIC phenotype, in which 100–1000 3D cultured TICs were capable of forming tumors in vivo [21–23]. To assess whether our 3D collagen gels could promote TIC proliferation, we examined the colony formation capability of cancer cells seeded in collagen gels. As shown in Figs. 1E and 3D collagen culture significantly strengthened the colony formation capability of SGC-7901 and BGC-823 cells (Fig. 1E), when compared with dish culture. Meanwhile, to determine the tumorigenic potential of 3D collagen cultured cells, 10^5^ SGC-7901 or BGC-823 cells (dish or 3D collagen culture) were subcutaneously injected into NOD-SCID mice (n = 10 in each group). The tumor diameter > 1 mm were determined as tumorigenicity. The percentage of mice with tumorigenicity in 10 mice was determined as tumorigenesis rate. Consistent to our hypothesis, greater tumorigenicity was observed in 3D collagen cultured gastric cancer cells (Fig. 1F). Subsequently, we sought to examine the TICs marker CD44 and CD133 in 3D cultured tumor cells by flow cytometry and quantified real-time PCR. Consistently, significant increase of CD44 and CD133 expression in 3D cultured SGC-7901/BGC-823 cells were observed, when compared with dish culture (Fig. 1G and Additional file 1: Fig. S1B). Additionally, elevated expression of stem cell factors *c-Myc* and *KlF4* was found in 3D cultured SGC-7901/BGC-823 cells. Thus, soft 3D collagen gels promoted the TIC phenotype in gastric cancer cells, which is consistent with the genomic expression profile shown in Fig. 1A. TICs with high tumorigenicity potential usually exhibit migratory and invasive characteristics, in addition to chemoresistance. Here, to determine the cell invasion and chemoresistance of TICs, SGC-7901/BGC-823 cells were collected from 3D collagen gels for Transwell and cell cytotoxicity assays (Annexin/PI staining). As anticipated, 3D collagen culture resulted in increased migratory properties (Fig. 1H) and resistance to chemotherapeutic 5-FU (Fig. 1I) and PTX (Fig. 1J), indicating that collagen promotes TIC migratory and chemo-resistant phenotypes. To further confirm the essential role of collagen in tumor progression, gastric tumor tissues were collected from patients, and the expression of collagen I was determined using Immunohistochemistry. Indeed, elevated expression of collagen I was found in tumor tissues from high stage patients (n = 10, stage T3–T4) compared to the low stage group (n = 10, stage T1–T2) (Fig. 1K). Together, those results suggest that collagen promotes TIC proliferation and positively correlates with the tumor progression in gastric cancer.

### Collagen gels mediate TICs characteristics through ITGB1 signaling in gastric cancer

**Fig. 2:**
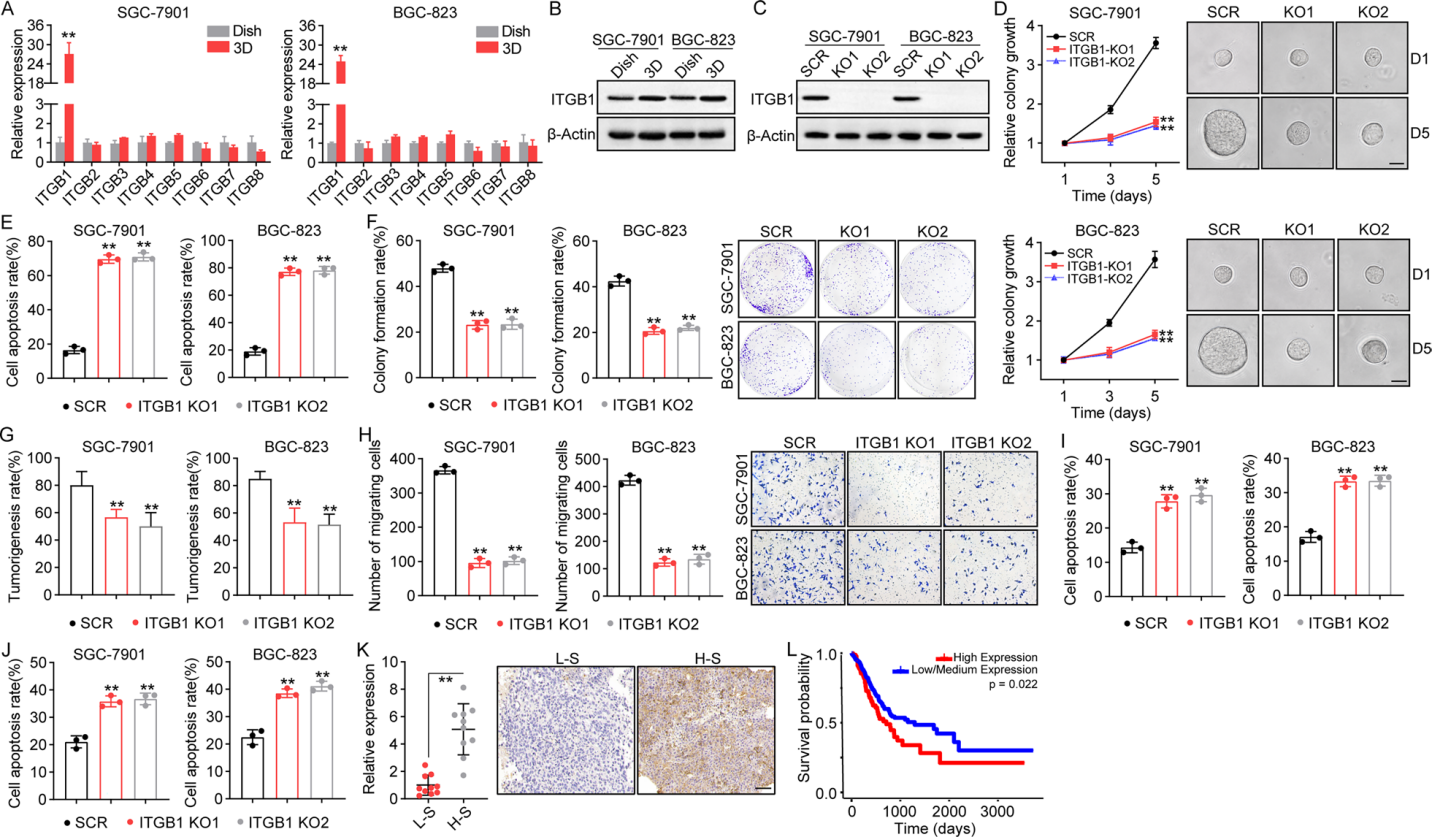
Collagen gels mediated TICs characteristics through ITGB1 signaling in gastric cancer.** A** Relative expression of ITGB1–8 in SGC-7901/BGC-823 cells cultured in dish or 3D gels, which was determined by real-time quantitative PCR.** B** Western blotting of ITGB1 in SGC-7901/BGC-823 cells cultured in dish or 3D gels.** C** Western blotting of ITGB1 in SGC-7901/BGC-823 (SCR) and ITGB1 KO SGC-7901/BGC-823 (KO1, KO2).** D** The colony growth curve of SGC-7901/BGC-823 and ITGB1 KO SGC-7901/BGC-823 cells in 3D collagen gels. The scale bar is 50 μm.** E** The cell apoptosis of SGC-7901/BGC-823 and ITGB1 KO SGC-7901/BGC-823 cells in 3D collagen gels on day 5.** F** SGC-7901/BGC-823 and ITGB1 KO SGC-7901/BGC-823 cells were cultured in 3D collagen gels for 5 days. Then tumor cells were collected and the colony formation capability was examined.** G** Tumor cells in (**F**) were collected and the tumorigenic capability was examined in mice. 10^5^ SGC-7901 or BGC-823 cells were subcutaneously injected into NOD-SCID mice (n = 10 in each group, day 30). The tumor diameter > 1 mm were determined as tumorigenicity. The percentage of mice with tumorigenicity in 10 mice was determined as tumorigenesis rate.** H** Tumor cells in (**F**) were collected and the cell migration was examined by transwell (24 h).** I** Tumor cells in (**F**) were collected and treated with 5-FU (1 µg/ml) for 48 h. The cell apoptosis was examined using Annexin/PI staining.** J** Tumor cells in (**F**) were collected and treated with PTX (2 µg/ml) for 48 h. The cell apoptosis was examined using Annexin/PI staining.** K** Immunohistochemical staining of ITGB1 in gastric tumor tissues from high stage (H-S, stage T3–4) and low stage (L-S, stage T1–2) patients (n=10 in each group). The scale bar is 50 μm.** L** Overall survival of gastric cancer patients with high/low ITGB1 expression (low expression, n= 95 and high expression, n= 297)

Components in the extracellular matrix, such as collagen I and fibronectin, have been demonstrated to mediate pro-survival signaling transduction through biomechanical force-associated integrin receptors [24]. Increasing evidence has suggested that integrins serve as transmembrane receptors, participating in cell-extracellular matrix adhesion and signal transduction [25, 26]. To assess the potential role of integrins in collagen-associated tumor progression, real-time quantitative PCR was performed to examine the expression of integrins in SGC-7901/BGC-823 cells cultured in a dish or in 3D gels. Intriguingly, both SGC-7901 and BGC-823 cells cultured in 3D collagen gels displayed elevated expression of ITGB1, when compared to the dish culture group (Fig. 2A). The similar result was confirmed at the protein level by western blotting (Fig. 2B), indicating that ITGB1 might be involved in gastric cancer progression induced by collagen. To further confirm our hypothesis, ITGB1 was knocked down in SGC-7901 and BGC-823 cells (Fig. 2C) and Annexin/PI staining cell apoptosis analysis revealed no obvious cell apoptosis in ITGB1 KO cells (Additional file 1: Fig. S2A). Subsequently, ITGB1 KO SGC-7901/BGC-823 cells were seeded in 3D collagen gels and colony growth was examined. In this experiment, the colony growth of tumor cells was significantly inhibited (Fig. 2D), and elevated cell apoptosis was observed in ITGB1 KO SGC-7901/BGC-823 cells (Fig. 2E). We next collected 3D cultured ITGB1 KO SGC-7901/BGC-823 cells and assessed their TIC phenotype. Consistently, ITGB1 knockdown suppressed colony formation (Fig. 2F) and tumorigenic (Fig. 2G) potential induced by collagen. Meanwhile, transwell and cell apoptosis analysis revealed that blockade of ITGB1 signaling resulted in cell migration inhibition (Fig. 2H) and chemoresistance suppression (Fig. 2I, J), indicating that collagen promotes TIC properties through ITGB1 dependent signaling in gastric cancer. To assess the specificity of the knockdowns, a rescue experiment was performed. The tumor suppressive effects of ITGB1 knockdown were reversed by ITGB1 overexpression (Additional file 1: Fig. S2B and C). Lastly, the expression of ITGB1 was detected in gastric tumor tissues from clinical patient by immunohistochemistry, in which tumor tissues from high stage patients (n = 10, stage T3–T4) revealed elevated ITGB1 expression compared to the low stage group (n = 10, stage T1–T2) (Fig. 2K). More importantly, the TCGA database further suggested poor overall survival in gastric cancer patients with high ITGB1 expression (Fig. 2L). Together, these results suggest that collagen promotes the TIC phenotype and gastric cancer progression through ITGB1 signaling.

### ITGB1 regulates TICs through YBX1/SPP1 signaling

**Fig. 3:**
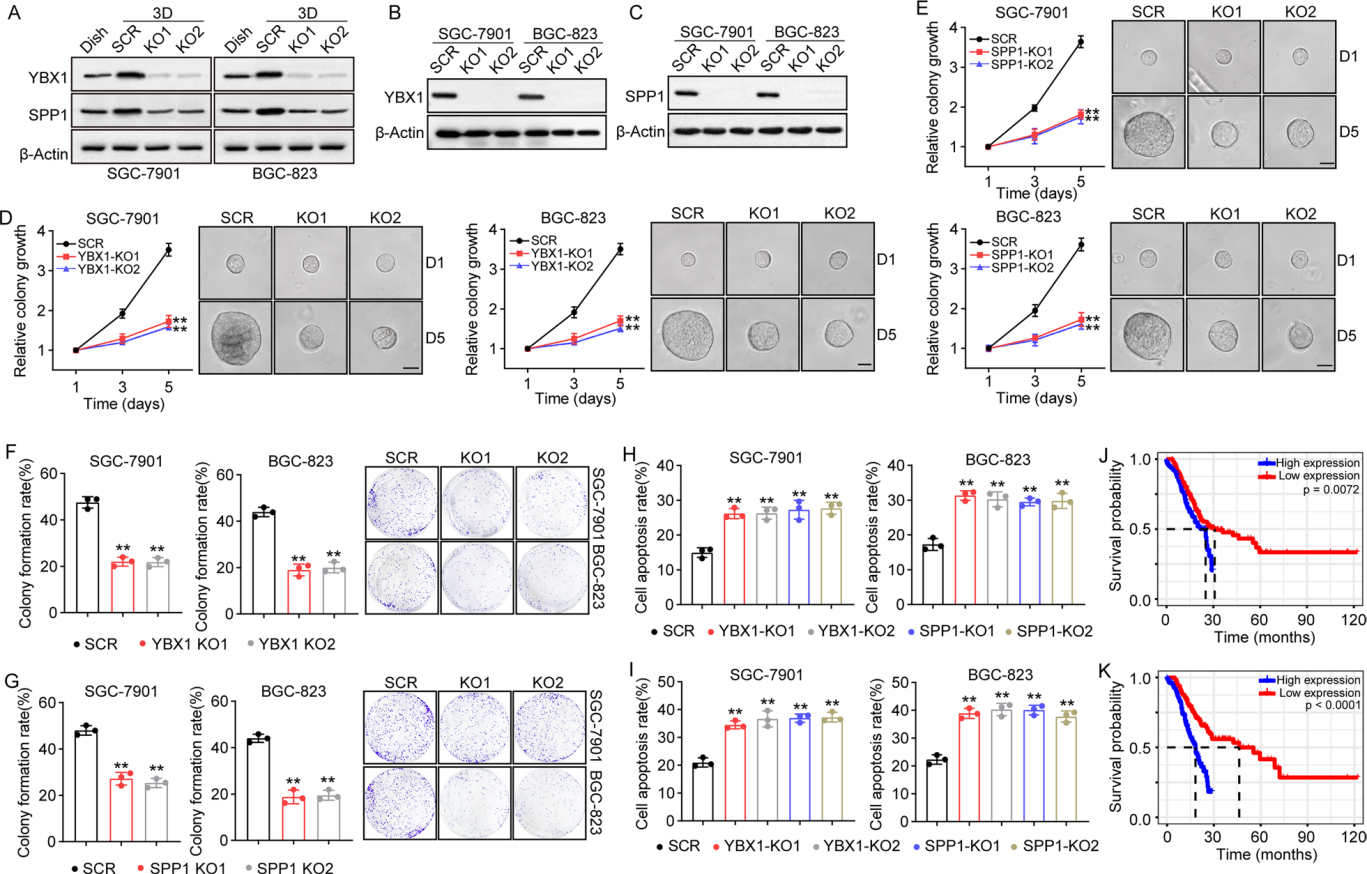
ITGB1 regulated TICs characteristics through YBX1/SPP1 signaling. **A** Western blotting of YBX1 and SPP1 in SGC-7901/BGC-823 (Dish), 3D cultured SGC-7901/BGC-823 and 3D cultured ITGB1 KO SGC-7901/BGC-823 cells (KO1, KO2). **B** Western blotting of YBX1 in SGC-7901/BGC-823 (SCR) and YBX1 KO SGC-7901/BGC-823 cells (KO1, KO2). **C** Western blotting of SPP1 in SGC-7901/BGC-823 (SCR) and SPP1 KO SGC-7901/BGC-823 cells (KO1, KO2). **D** The colony growth curves of SGC-7901/BGC-823 and YBX1 KO SGC-7901/BGC-823 cells in 3D collagen gels. The scale bar is 50 μm. **E** The colony growth curves of SGC-7901/BGC-823 and SPP1 KO SGC-7901/BGC-823 cells in 3D collagen gels. The scale bar is 50 μm. **F** The colony formation capability of SGC-7901/BGC-823 and YBX1 KO SGC-7901/BGC-823 cells in dish. **G** The colony formation capability of SGC-7901/BGC-823 and SPP1 KO SGC-7901/BGC-823 cells in dish. **H** SGC-7901/BGC-823, YBX1 KO SGC-7901/BGC-823 and SPP1 KO SGC-7901/BGC-823 cells were collected and treated with 5-FU (1 µg/ml) for 48 h. The cell apoptosis was examined using Annexin/PI staining. **I** SGC-7901/BGC-823, YBX1 KO SGC-7901/BGC-823 and SPP1 KO SGC-7901/BGC-823 cells were collected and treated with PTX (2 µg/ml) for 48 h. The cell apoptosis was examined using Annexin/PI staining. **J** overall survival of gastric cancer patients with high/lowYBX1 expression (low expression, n = 173 and high expression, n = 172). **K** overall survival of gastric cancer patients with high/low SPP1 expression (low expression, n = 169 and high expression, n = 172)

Previous studies have demonstrated that integrins bind YBX1 to mediate the activation of pro-survival signaling pathways in cancer cells [27]. Additionally, YBX1 is capable of interacting with G3BP1 to promote SPP1 upregulation, resulting in renal cancer invasion and metastasis [28]. Intriguingly, our western blotting showed obvious overexpression of SPP1 in gastric tumor tissues in the TCGA database, when compared to normal tissues (Fig. 1A). This indicates that ITGB1 might participate in gastric cancer progression through YBX1/SPP1 signaling. Herein, we examined the expression of YBX1 and SPP1 in collagen cultured SGC-7901/BGC-823 cells by western blotting. And elevated expression of YBX1 and SPP1 was observed in 3D cultured groups. Importantly, the upregulation of YBX1 or SPP1 was reversed in ITGB1 KO SGC-7901/BGC-823 cells (Fig. 3A). Therefore, we confirmed that 3D collagen gels stimulate elevated expression of YBX1 and SPP1 through ITGB1 in gastric cancer. Subsequently, we further established YBX1 knockdown (Fig. 3B) and SPP1 knockdown (Fig. 3C) SGC-790/BGC-823 cells, and determined whether inhibition of YBX1/SPP1 signaling affected the TIC phenotype in 3D collagen gels. No obvious cell apoptosis was observed in YBX1 knockdown (Additional file 1: Fig. S3A) and SPP1 knockdown (Fig. S3B) SGC-7901/BGC-823 cells. Meanwhile, blockade of YBX1 and SPP1 suppressed colony growth in 3D gels (Fig. 3D, E). Meanwhile, 3D cultured YBX1/SPP1 silenced gastric cancer cells showed a weakened ability to form colonies (Fig. 3F, G) as well as diminished chemoresistance (Fig. 3H, I), indicating that 3D collagen promotes the TIC phenotype through YBX1 and SPP1. To further confirm the role of YBX1 and SPP1 in gastric cancer development, we assessed overall survival in gastric cancer patients with low/high expression of YBX1 and SPP1 using TCGA database analysis. Consistently, both YBX1 (Fig. 3 J) and SPP1 (Fig. 3K) negatively correlated with the survival time of patients. These data provide evidence that collagen regulates the TIC characteristics of gastric cancer cells by ITGB1/YBX1/SPP1 signaling.

### SPP1 activates NF-κB signaling to promote gastric cancer progression

**Fig. 4:**
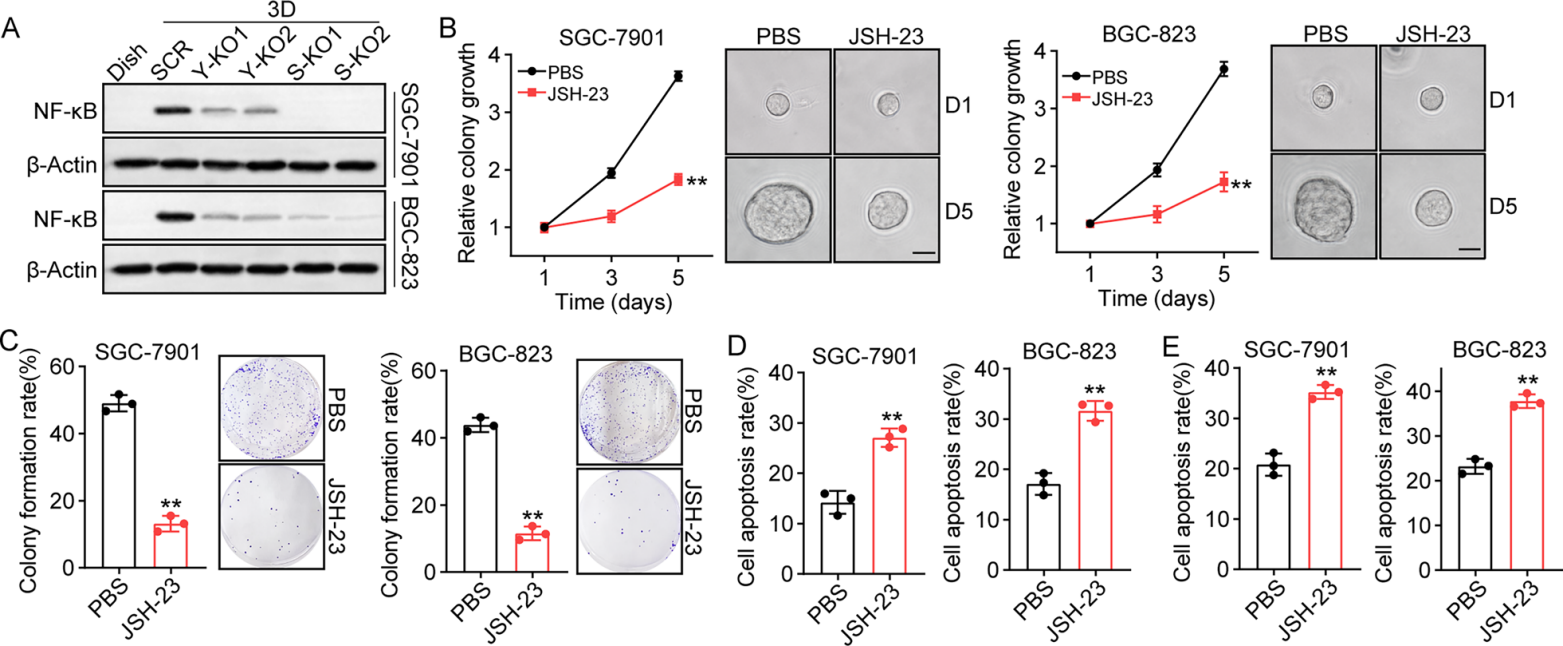
SPP1 activated NF-κB signals to promote gastric cancer progression. **A** Western blotting of NF-κB in SGC-7901/BGC-823 (Dish), 3D cultured SGC-7901/BGC-823 (SCR), 3D cultured YBX1 KO SGC-7901/BGC-823 (Y-KO1, Y-KO2) and 3D cultured SPP1 KO SGC-7901/BGC-823 cells (S-KO1, S-KO2). **B** SGC-7901/BGC-823 cells were cultured in 3D collagen gels and treated with PBS or JSH-23 (50 nM). The colony growth was examined. The scale bar is 50 μm. **C** SGC-7901/BGC-823 cells in **B** were collected and the colony formation capability was examined in dish. **D** SGC-7901/BGC-823 cells in **B** were collected and treated with 5-FU (1 µg/ml) for 48 h. The cell apoptosis was examined using Annexin/PI staining. BE SGC-7901/BGC-823 cells in **B** were collected and treated with PTX (2 µg/ml) for 48 h. The cell apoptosis was examined using Annexin/PI staining

NF-κB is a transcription factor crucial to inflammatory responses and cancer progression. Increasing evidence has suggested that NF-κB signaling is capable of promoting stem-like phenotypes and drug resistance in a series of cancer types [29]. Thus, we sought to verify whether YBX1/SPP1 mediated gastric cancer development through NF-κB signaling. Here, we firstly examined the expression of NF-κB in gastric cancer cells by western blotting. 3D collagen cultured SGC-7901/BGC-823 cells exhibited enhanced expression of NF-κB, when compared to dish cultured cells. Moreover, suppression of YBX1 or SPP1 suppressed the activation of NF-κB in gastric cancer cells cultured in 3D gels (Fig. 4A), indicating that 3D collagen culture upregulated NF-κB expression through YBX1/SPP1 signaling. In addition, treatment with the NF-κB inhibitor JSH-23 retarded the colony growth of SGC-7901/BGC-823 cells in 3D collagen gels (Fig. 4B). JSH-23 treated cells exhibited a weakened ability to form colonies (Fig. 4C), as well as attenuated chemoresistance (Fig. 4D, E). Together, these results suggest that 3D collagen regulates TIC proliferation through ITGB1/YBX1/SPP1/ NF-κB signaling.

### Blockade of ITGB1/NF-κB signaling improves outcomes in gastric cancer therapy

**Fig. 5:**
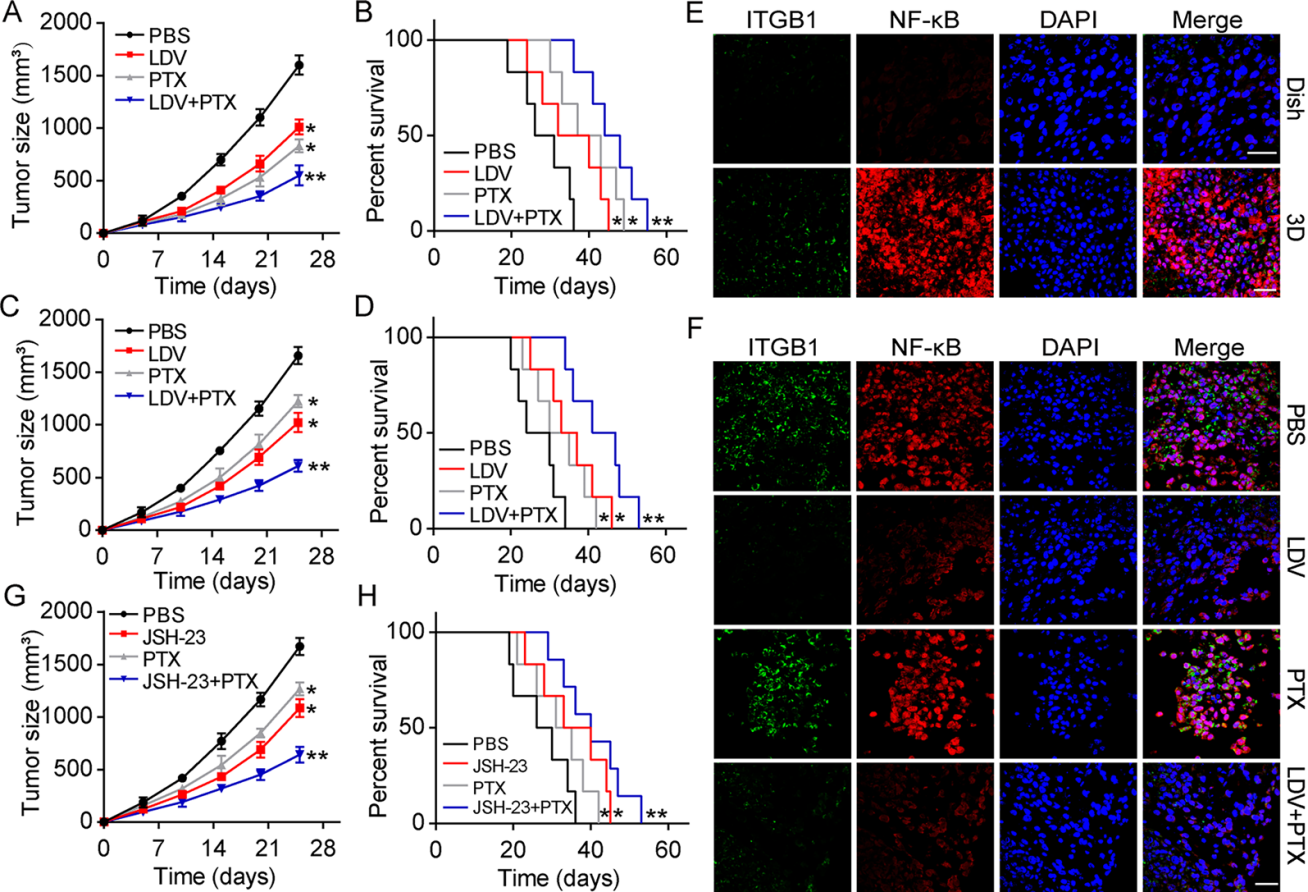
Blockade of ITGB1/ NF-κB signals revealed improved outcome in gastric cancer therapy. **A** Tumor volumes of SGC-7901 bearing mice treated with PBS, LDV, PTX and LDV combining PTX. **B** Survival time of SGC-7901 bearing mice treated with PBS, LDV, PTX and LDV combining PTX. **C** Tumor volumes of 3D collagen cultured SGC-7901 bearing mice treated with PBS, LDV, PTX and LDV combining PTX. **D** Survival time of 3D collagen cultured SGC-7901 bearing mice treated with PBS, LDV, PTX and LDV combining PTX. **E** Immunofluorescence of ITGB1 and NF-κB in tumor tissues from SGC-7901 bearing mice and 3D collagen cultured SGC-7901 bearing mice on day 15. The scale bar is 50 μm. **F** immunofluorescence of ITGB1 and NF-κB in tumor tissues from 3D collagen cultured SGC-7901 bearing mice on day 30 (treated with PBS, LDV, PTX, LDV combining PTX). The scale bar is 50 μm. **G** Tumor volumes of 3D collagen cultured SGC-7901 bearing mice treated with PBS, JSH-23, PTX and JSH-23 combining PTX. **H** Survival time of 3D collagen cultured SGC-7901 bearing mice treated with PBS, JSH-23, PTX and JSH-23 combining PTX

Both clinical trials and experimental evidence has suggested that conventional chemotherapeutic interventions, such as 5-FU and PTX, fail to eliminate tumor cells due to drug resistance induced by TICs [30]. Studies have also provided evidence to suggest that inhibition of TICs within tumor tissues could significantly improve outcomes in clinical treatment [31]. Our previous data showed that the activation of ITGB1/NF-κB signaling elicits a TIC-like phenotype in gastric cancer cells, eventually resulting in the occurrence of chemoresistance. To eliminate TICs and reverse chemoresistance in gastric cancer, we explored the combination of the ITGB1 inhibitor LDV and the NF-κB inhibitor JSH-23 along with a chemotherapeutic agent to suppress tumor growth. Here, we established subcutaneous SGC-7901-bearing mice and treated mice with PBS, LDV, PTX and LDV combined with PTX when tumors reached 700 mm^3^. Notably, the combination of PTX and the ITGB1 inhibitor LDV significantly suppressed tumor growth (Fig. 5A) and prolonged the overall survival of tumor-bearing mice (Fig. 5B). Next, we wondered whether combining treatment could reverse drug resistance induced by collagen. Thus, we further established subcutaneous tumor bearing mice using 3D collagen cultured SGC-7901 cells, and treated mice with PTX combined with LDV. Intriguingly, PTX exhibited limited tumor suppressive effects in 3D collagen cultured SGC-7901 bearing mice, which might have been caused by drug resistance induced by collagen/ITGB1 signaling. However, LDV treatment significantly improved the anticancer effects of PTX and reversed chemoresistance in tumor-bearing mice (Fig. 5C, D). To confirm our hypothesis, we isolated those tumor tissues and examined the expression of ITGB1 and NF-κB. Intriguingly, 3D collagen cultured SGC-7901 cells exhibited elevated expression of ITGB1 and NF-κB compared to dish cultured SGC-7901 cells in vivo (Fig. 5E). LDV treatment obviously suppressed the activation of ITGB1/NF-κB signaling in tumor tissues, with or without PTX (Fig. 5F). These results indicate that activation of ITGB1 signaling is associated with chemoresistance development in tumor tissues, and suppression of ITGB1 signaling could help to strengthen the tumor cytotoxicity of therapeutic agents. Consistently, similar effects were observed with the combination of PTX and the NF-κB inhibitor JSH-23 (Fig. 5G, H). Taken together, these results support the notion that a combination of ITGB1/NF-κB inhibitors and chemotherapy could serve as an efficient strategy to reverse chemotherapy resistance and improve anticancer effects in gastric cancer therapy.

## Discussion

Clinical evidence and experimental data have shown that residual TICs are prone to eliciting tumor recurrence after surgical resection or chemotherapy [32]. However, the underlying mechanism of TIC generation and proliferation remains elusive. In this study, we provided evidence that extracellular collagen was able to dedifferentiate bulk cancer cells into TICs. The acquired TIC-like phenotypes of cancer cells were associated with the activation of ITGB1/YBX1/SPP1/NF-κB signaling. Those data provide additional support for the cancer stem cell hypothesis and insight into cell differentiation in tumor biology.

Increasing evidence has shown that differentiated tumor cells can revert into stem-like or tumor initiating cancer cells to promote tumor progression [33, 34]. Those studies strengthen the concept that tumor cells can be endowed with a TIC phenotype under specific environmental cues. In fact, compelling findings have reported that 3D fibrin gels can promote the selection of tumor repopulating cells, with a high potential for tumorigenicity after 3D culture [35, 36]. Meanwhile, both Matrigel and collagen gels have been demonstrated to stimulate the stem-like phenotypes of cancer cells in in specific tumor types, such as breast cancer [37]. Intriguingly, elevated expression of collagen was observed in gastric tumor tissues from TCGA database and clinical specimens. Nonetheless, high concentration 3D collagen gels described in previous studies (>1 mg/ml) failed to induce gastric cancer cell proliferation and colony formation. Despite the fact that collagen serves as the major element of the extracellular matrix, such a high concentration of collagen (>1 mg/ml) does not exist in the microenvironment of tumor tissues, so we reduced the collagen concentration and cultured gastric cancer cells in clotty collagen gels. Consequently, our improved 3D collagen gels (0.5 mg/ml) succeeded in sustaining the long-term viability of gastric cancer cells and promoted the TIC phenotype in vitro. Our results in combination with previous reports provide strong evidence that bulk cancer cells are plastic and able to revert into stem-like TICs under certain circumstances. Collagen is tightly involved in the TICs remodeling process. These results indicate the potential role of the extracellular matrix in TIC generation and provide novel insight into the underlying mechanism of TIC generation.

In our 3D collagen gels, gastric cancer cells displayed a spherical colony morphology, as well as TIC-like phenotypes. Previous documents have clarified that elements in the extracellular matrix, such as collagen and fibronectin, participate in the regulation of tumor stemness through integrin-associated receptors [38]. It has been demonstrated that cell adhesion to collagen occurs via integrin β1 or β3 [39]. Indeed, we found that gastric cancer cells in collagen gels exhibited dramatic activation of ITGB1 signaling, and ITGB1 silencing suppressed the TIC phenotype in tumor cells. Both ITGB1 and ITGB3 have been demonstrated to mediate tumor growth and promote tumor stemness in several tumor types [40, 41]. In fact, elevated expression of integrins also serves as a marker in TICs, i.e. integrin β3 in melanoma [42] and integrin β4 in lung cancer [43]. Mechanistically, integrins are tightly involved in pro-survival signal activation in tumor cells. Smiraglia provided evidence that integrin β5 can promote the tumorigenic potential of breast cancer cells through a Src-FAK and MEK-ERK associated signaling pathway [44]. Yan and colleagues demonstrated integrin β6-mediated lung cancer cell proliferation and distant metastasis through an IL-8 associated MAPK/ERK signaling pathway [45]. These results indicate that extracellular collagen might promote pro-survival signaling pathway activation through ITGB1 signaling. Compelling reports have implicated that YBX1 is activated by integrin signaling and participates in sustained tumor growth in bladder cancer [46]. Importantly, our genomic expression profiles suggested that gastric tumor tissues exhibit aberrant overexpression of SPP1, which has been shown to be upregulated by YBX1 in renal cancer cells [28]. In line with those observations, we found that 3D collagen significantly upregulated ITGB1 signaling, which further contributed to YBX1/SPP1/NF-κB signaling activation. In contrast with previous reports, we also confirmed that the ITGB1/YBX1/SPP1/NF-κB signaling axis is correlated with drug resistance development in gastric cancer, and blockade of ITGB1/NF-κB signaling led to improved anticancer effects in a subcutaneous gastric cancer mouse model. This finding highlights the role of ITGB1/NF-κB signaling in regulating the TIC phenotype in cancer cells, and provides a novel target for chemoresistance in clinical gastric cancer treatment.

Given the limitations of previous findings, our study further clarifies the essential role of collagen in regulating the TIC phenotype. Firstly, we identified the role of collagen in promoting gastric cancer progression, demonstrating that elevated expression of collagen also underscored the relevance of the extracellular matrix in gastric cancer development. Based on these observations, we described an improved 3D collagen gel for gastric cancer cell culture in vitro, which obviously promoted the TIC phenotype in cancer cells. Secondly, we further elucidated the underlying mechanism of collagen-induced TIC proliferation in gastric cancer. Our results demonstrate that 3D collagen culture regulated the TIC phenotype through ITGB1/YBX1/SPP1/NF-κB signaling. Thirdly, we provided a new strategy to reverse the drug resistance induced by TICs, in which ITGB1/NF-κB inhibitors combined with chemotherapy exhibited superior tumor suppressive effects in gastric tumor models. Finally, collagen and associated signaling molecules, including ITGB1, YBX1, SPP1 and NF-κB in tumor tissues, could serve as potential biomarkers to monitor tumor progression and predict chemoresistance.

## Conclusions

In summary, our results demonstrate that type I collagen is capable of promoting the TIC phenotype in gastric cancer cells via the ITGB1/YBX1/SPP1/NF-κB signaling pathway. ITGB1/NF-κB targeted therapy might provide innovative inroads into gastric cancer treatment.

## Supplementary Information

**Additional file 1.** Supplementary information.

## Data Availability

The anonymized data used and/or analyzed during the current study are available from the corresponding author on reasonable request.

## Abbreviations

TICs: Tumor initiating cells
ITGB1: Integrin β1
YBX1: Y-box binding protein 1
SPP1: Secreted phosphoprotein 1
ATCC: American Type Culture Collection
5-FU: 5 Fluorine
PTX: Paclitaxel
