# Supplementary material for: Promotion of gastric tumor initiating cells in a 3D collagen gel culture model via YBX1/SPP1/NF-κB signaling
Source: Cancer Cell Int. 2021 Nov 10;21:599. doi: 10.1186/s12935-021-02307-x (PMC8579534; doi:10.1186/s12935-021-02307-x)
Supplement: Supplementary file 1 — Additional file 1. Supplementary information. [file 12935_2021_2307_MOESM1_ESM.docx]

**Supplementary Material**


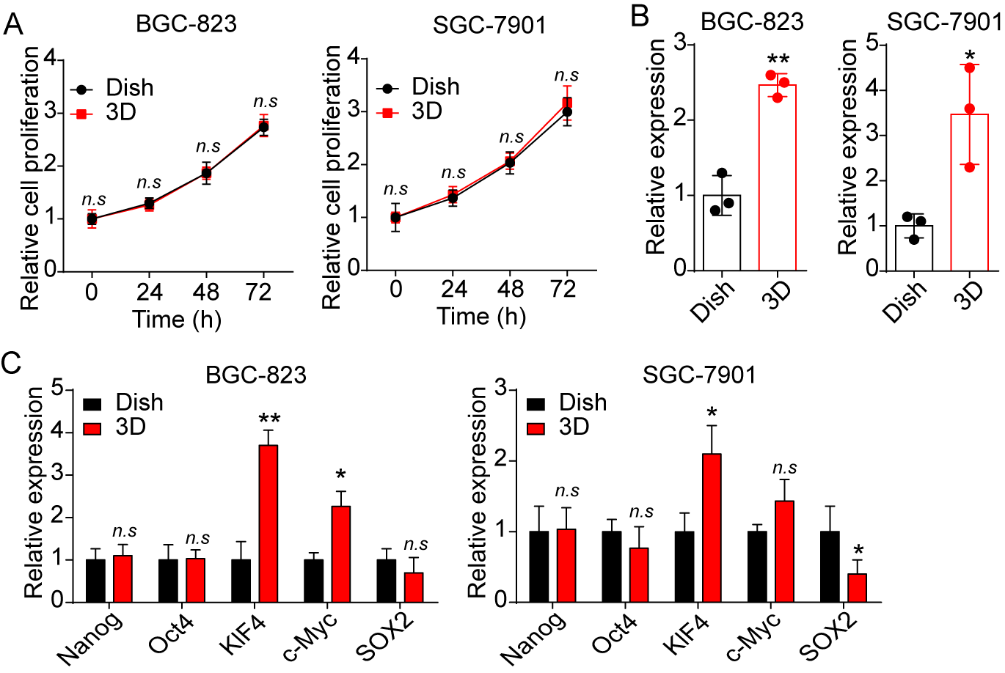


Supplementary figure 1

A, Dish or 3D collagen cultured BGC-823/SGC-7901 cells were collected and seeded in flask. The cell proliferation was determined using CCK-8 analysis. B, the CD133 expression of SGC-7901/BGC-823 cultured in dish or 3D collagen gels, which was determined by flow cytometry. C, relative *Nanog*, *Oct4*, *KlF4*, *c-Myc* and *SOX2* expression in SGC-7901/BGC-823 cultured in dish or 3D collagen gels, which was determined by quantified real-time PCR.


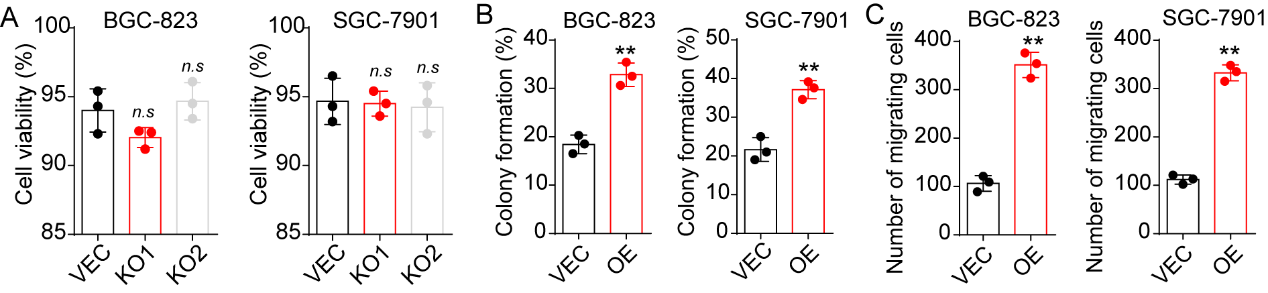


Supplementary figure 2

A, cell viability of vector or ITGB1 knockdown BGC-823/SGC-7901. B, ITGB1 was overexpressed again in ITGB1 knockdown BGC-823/SGC-7901 cells. Then the colony formation capability was determined. C, ITGB1 was overexpressed again in ITGB1 knockdown BGC-823/SGC-7901 cells. Then cell migration was examined by transwell assay.


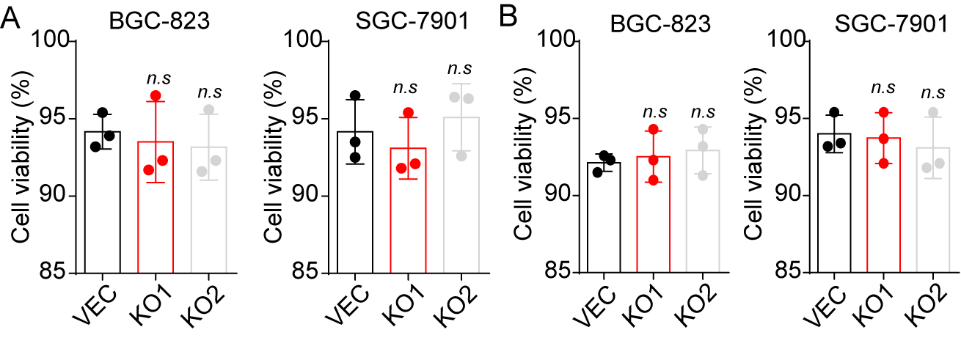


Supplementary figure 3

A, cell viability of vector or YBX1 knockdown BGC-823/SGC-7901. B, cell viability of vector or SPP1 knockdown BGC-823/SGC-7901.
